# The Bioinformatic Analysis of the Dysregulated Genes and MicroRNAs in Entorhinal Cortex, Hippocampus, and Blood for Alzheimer's Disease

**DOI:** 10.1155/2017/9084507

**Published:** 2017-11-21

**Authors:** Xiaocong Pang, Ying Zhao, Jinhua Wang, Qimeng Zhou, Lvjie Xu, De Kang, Ai-Lin Liu, Guan-Hua Du

**Affiliations:** ^1^State Key Laboratory of Bioactive Substance and Function of Natural Medicines, Institute of Materia Medica, Chinese Academy of Medical Sciences and Peking Union Medical College, Beijing 100050, China; ^2^Beijing Key Laboratory of Drug Target and Screening Research, Beijing 100050, China

## Abstract

**Aim:**

The incidence of Alzheimer's disease (AD) has been increasing in recent years, but there exists no cure and the pathological mechanisms are not fully understood. This study aimed to find out the pathogenesis of learning and memory impairment, new biomarkers, potential therapeutic targets, and drugs for AD.

**Methods:**

We downloaded the microarray data of entorhinal cortex (EC) and hippocampus (HIP) of AD and controls from Gene Expression Omnibus (GEO) database, and then the differentially expressed genes (DEGs) in EC and HIP regions were analyzed for functional and pathway enrichment. Furthermore, we utilized the DEGs to construct coexpression networks to identify hub genes and discover the small molecules which were capable of reversing the gene expression profile of AD. Finally, we also analyzed microarray and RNA-seq dataset of blood samples to find the biomarkers related to gene expression in brain.

**Results:**

We found some functional hub genes, such as* ErbB2*,* ErbB4*,* OCT3*,* MIF*,* CDK13*, and* GPI*. According to GO and KEGG pathway enrichment, several pathways were significantly dysregulated in EC and HIP.* CTSD *and* VCAM1* were dysregulated significantly in blood, EC, and HIP, which were potential biomarkers for AD. Target genes of four microRNAs had similar GO_terms distribution with DEGs in EC and HIP. In addtion, small molecules were screened out for AD treatment.

**Conclusion:**

These biological pathways and DEGs or hub genes will be useful to elucidate AD pathogenesis and identify novel biomarkers or drug targets for developing improved diagnostics and therapeutics against AD.

## 1. Introduction

Alzheimer's disease (AD) is the most common form of dementia related to age, accounting for 50%–60% of all cases and is characterized by a progressive decline in memory associated with other cognitive deficits: judgement, abstraction, language, attention, and visuoconstructive abilities [1]. Approximately 36 million people were affected by AD worldwide, and it is estimated that, by 2050, the number of cases will rise to 110 million [2]. However, there is no cure for AD and the pathological mechanisms of AD are not fully understood now.

Genetic linkage analyses of familial cases have led to the identification of causative mutations in three genes,* APP*,* PSEN1*, and* PSEN2*, as well the identification of a high-risk factor: the E4 allele of* APOE* [3]. More recently, several independent genome-wide association studies (GWAS) identified 21 new genetic loci by* CLU*,* PICALM*,* CR1*,* BIN1*,* CD33*,* ABCA7, MS4A6A*,* MS4A4E*,* CD2AP*,* EPHA1*,* HLA-DRB5/DRB1*,* SORL1*,* PTK2B*,* SLC24A4*,* ZCWPW1*,* CELF1*,* FERMT2*,* CASS4*,* INPP5D*,* MEF2C*,* and NME8* [3]. But most genes affect A*β* production and clearance. It remains to be seen whether additional pathways are identified or whether most genes will fall into the already identified pathways and cellular mechanisms [4].

Brain regions show different susceptibilities to the pathological and metabolic characteristics of AD [5]. Entorhinal cortex (EC) and hippocampus (HIP) are thought to be important regions in differentiating AD from normal aging, and the earliest neuropathological changes in AD appear in EC and then progress to HIP [6, 7]. Furthermore, memory and learning formation depends on the presence of an intact entorhinal–hippocampal circuit [8]; therefore analyzing gene expression of EC and HIP is beneficial for identifying biological pathways related to memory and learning perturbed in AD.

**Figure 1:**
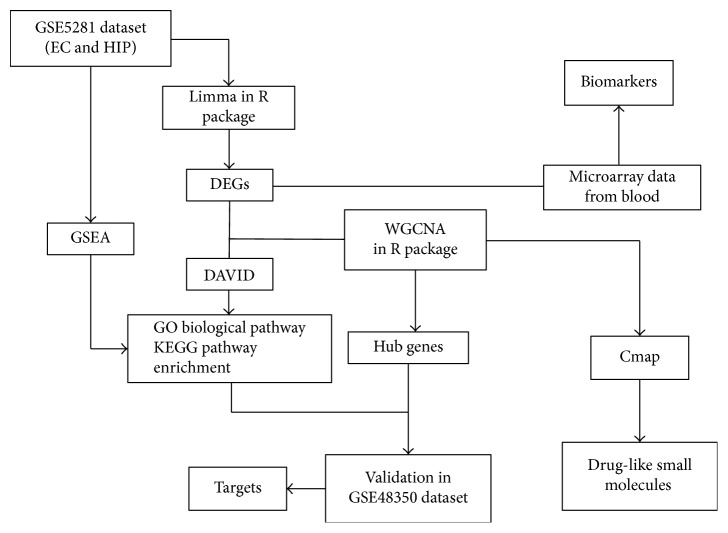
The workflow for analysis of differentially expressed genes (DEGs) and finding out the pathogenesis of learning and memory impairment, new biomarkers, potential therapeutic targets, and drugs for AD.

In this study, our objective is to find out the pathogenesis of learning and memory impairment, new biomarkers, potential therapeutic targets, and drugs for AD. The workflow was summarized in Figure 1. The gene expression profiles on AD and control (CT) samples from Gene Expression Omnibus (GEO) database [9] were downloaded, and then the DEGs in EC and HIP regions were analyzed for functional and pathway enrichment analysis of Gene Ontology (GO) and Kyoto Encyclopedia of Genes and Genomes (KEGG) to elucidate the pathological mechanism of AD in EC and HIP regions. We also utilized the DEGs in EC and HIP to construct coexpression networks to identify hub genes, and then these genes were uploaded to Connectivity Map (CMAP) [10] to discover the small molecules which were capable of reversing the gene expression profile of AD. Blood has the property of easy accessibility, sufficiently high specificity and sensitivity, and low costs [11]. Therefore, we further analyzed the DEGs in the blood samples of microarray and RNA-seq dataset to find the biomarkers in blood and the relationship with gene expression in brain, which could provide important information for AD diagnosis and therapy.

## 2. Methods

### 2.1. Gene Expression Profiles of AD

Gene microarray technology allows massively parallel analysis of most genes expressed in a tissue. The microarray data used in the study were obtained from GEO database [12]. The primary dataset, containing expression data of EC and HIP, was downloaded from GEO database (GEO Accession Number: GSE5281) [13] including 10 AD and 13 CT samples. GSE5281 was selected based on rational experiment design with a very good quality and reliability and provided plenty of information for data mining [14, 15]. The platform was GPL570 (Affymetrix Human Genome U133 Plus 2.0 Array). To validate of findings from GSE5281, we used another gene expression data of EC and HIP from GSE48350, which is including 39 CT and 15 AD samples in HIP and 42 CT and 19 AD samples in EC, respectively [16].

Gene expression profiles of peripheral blood mononuclear cells were obtained from GSE4226 [17], which included 14 normal elderly controls (NEC) and 14 AD subjects to find the potential biomarker in blood sample. MicroRNAs (miRNAs) have also demonstrated their potential as noninvasive biomarkers from blood for a wide variety of human pathologies. Therefore, noncoding RNA profiling by high throughput sequencing from GSE46579 [18] and expression profiling by RT-PCR from GSE90828 [19] were analyzed for plasma microRNA biomarker. The blood samples of GSE90828 were collected from 30 age-matched controls (normal, 12 males and 18 females, mean age of 70.4) and 23 MCI (whole name) patients (11 males and 12 females, mean age of 72.8), and blood samples of GSE46579 included 48 AD patients and 22 unaffected controls.

### 2.2. Data Preprocessing and Differential Gene Analysis

The original expression profiles in CEL (whole name) format of GSE5281, GSE48350, GSE4226, and GSE90828 were transformed into a matrix using affy package in R language. The median method was used for normalizing the expression matrix. Subsequently, the Limma package was utilized to identify the differential genes between the AD and CTs. The threshold for the *P* value was set to 0.05 and |log_2_⁡FC| was set to 1. Differential expression analysis of GSE46579 was conducted using edgeR with a threshold of the *P* value ≤ 0.05 and an absolute value of log_2_⁡FC ≥ 1 being used to judge the significance of the gene expression differences.

### 2.3. Weighted Gene Coexpression Network Analysis (WGCNA)

WGCNA was performed in R using the WGCNA package [20]. The EC and HIP regions microarray data of GSE5281 was considered as a primary source for the analysis. The network construction started by calculating robust correlations between all genes across all relevant samples. The correlation adjacency matrix was increased to the power *β* = 16 based on scale-free topology criterion. The power parameter was selected to amplify the strong connections between genes and penalize the weaker connections.

The first principle component was considered as the module eigen gene (ME), which was representing the highest percent of variance for all the genes in a module. Module membership (kME) measured the correlations between each gene and each ME. The within-module connectivity (kin) for each gene was determined by summing the connectivity of that gene with each other gene set in that module [21, 22]. Genes, which have significant correlations with MEs and high within-module connectivity, were considered as hub genes of the modules. The hub genes were confirmed using Cytoscape's cytoHubba plugin [23].

### 2.4. GO Biological Pathway and KEGG Pathway Enrichment Analysis

We extracted the DEGs of EC and HIP for GO biological pathway and KEGG pathway enrichment. GO provides a useful tool to look for the common traits that are shared within a list of genes, which are represented by the GO terms associated with a large portion of the genes in the gene list [24]. KEGG is a useful online pathway archive that allows experimental data detailing the molecular functions of proteins to be organized in a useful, consistent format that supports computational mining and querying [25]. The Database for Annotation, Visualization and Integrated Discovery (DAVID) consists of an integrated biological knowledgebase and analytic tools aimed at systematically extracting biological meaning from large gene lists to assist investigators to annotate remarkable genes of specific function [26]. In this study, DEGs were subjected to GO and KEGG analysis with DAVID Bioinformatics Resources 6.7. EASE score (or called *P* value) of 0.05 was used as cutoff criteria.

Gene Set Enrichment Analysis (GSEA) was also applied to identify significant pathways in GSE5281 and GSE48350 based on GO Biological Process (GO_BP) and KEGG pathway. This method specified whether the pathways were randomly distributed at the top or bottom of the detected genes. The coefficients of Spearman correlation between genes and sample label were defined as the weight of genes [27]. Statistical significance was assessed by comparing the enrichment score to enrichment results generated from 1000 random permutations of the gene sets to obtain *P* values (nominal *P* value). The significant level of pathways was considered with levels of FDR ≤ 0.1 and *P* ≤ 0.05. FunRich 2.1.2 was used to compare the varied genes in blood and in EC or HIP by GO_terms.

### 2.5. Screening of Drug-Like Small Molecules

The hub genes in the interaction network were divided into two groups of upregulated and downregulated genes. By comparing the expression pattern similarities of the differential genes and genes perturbed by small molecules in the CMAP (https://www.broadinstitute.org/cmap/#) [28], small molecules involved in the disease were identified. Small molecules with a score >0.7 were considered to be associated with the disease.

## 3. Results

### 3.1. Screening of DEGs

**Figure 2:**
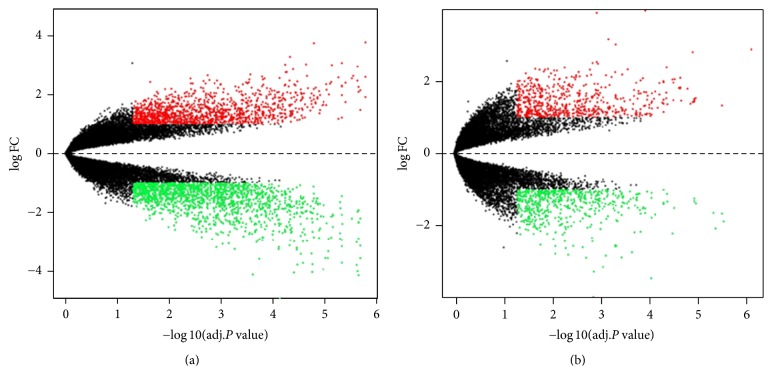
The volcano plots for EC (a) and HIP (b). The red and green spots represent upregulated and downregulated DEGs, respectively.

To extract the gene expression data (GSE5281) on patients with AD compared with CTs from GEO database, we utilized Limma R package to analyze DEGs between 10 AD and 13 CT samples. According to the cutoff criteria, 3008 DEGs including 1365 upregulated and 1643 downregulated DEGs were identified in EC, while 1232 DEGs including 638 upregulated and 594 downregulated DEGs were identified in HIP (Figure 2). In addition, 283 overlapping DEGs were found in the two regions.

### 3.2. Dysfunctional Coexpression Network Construction

**Figure 3:**
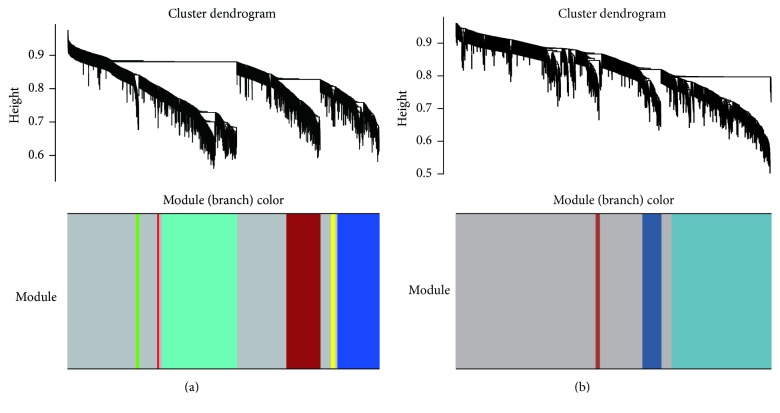
Dendrograms produced by average linkage hierarchical clustering of genes based on topological overlaps in the GSE5281. The extent of gene conservation in the datasets was represented by the same module colors.

**Figure 4:**
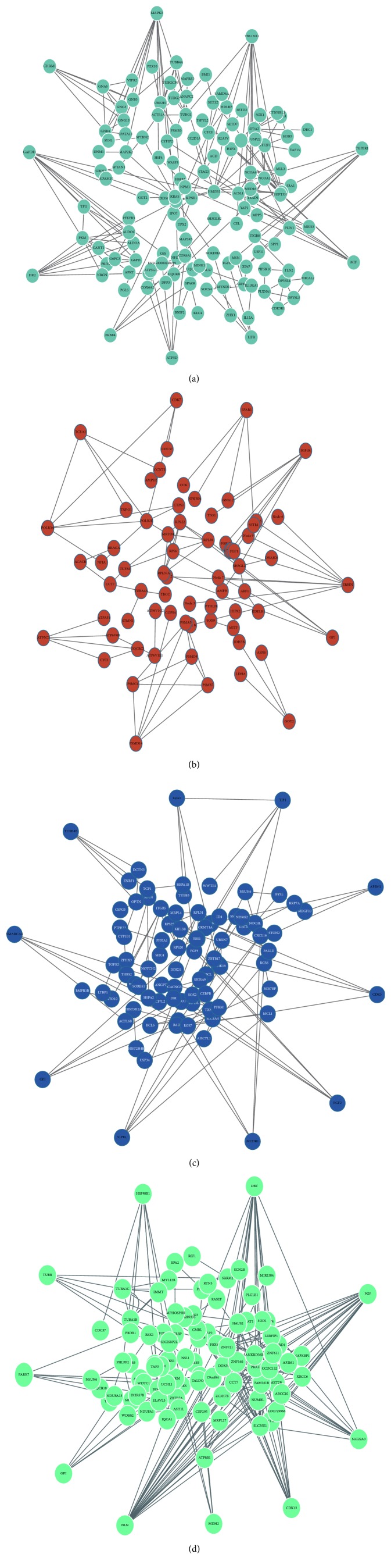
Network visualization of the top modules identifies AD hub genes. Based on WGCNA convection the top three enriched modules of EC were named as turquoise (a), brown (b), and blue (c), and there was an enriched module of HIP, named as turquoise (d). The AD specific hub genes in each module were ranked in larger circle.

WGCNA was used utilizing the coexpression of the DEGs. The module preservation function evaluates the module preservation by implementing various network based statistics. *Z*-summary is one such statistic measure summarizing the composite preservation. The *Z*-summary > 10 indicated evidence of strong preservation of the modules across all the datasets. Based on WGCNA convection, the top three enriched modules of EC were named as turquoise, brown, and blue, and there was one enriched module of HIP, named as turquoise (Figure 3). In the study, adjacency cutoff value of WGCNA was set to 0.75 so that a relatively large number of nodes could be retained in coexpression network and the accuracy of prediction of relationship of DEGs can be ensured at the same time. Degree, the topological parameter which determines the connectedness between the nodes, was chosen as the parameter for hub gene selection. The top 10 genes with high degree identified by WGCNA in all the modules were reported as hubs by cytoHubba. Finally, two separate coexpression networks of EC and HIP were built and visualized by Cytoscape 3.2.1 (Figure 4).

### 3.3. Gene Ontology and Pathway Enrichment Analysis

**Figure 5:**
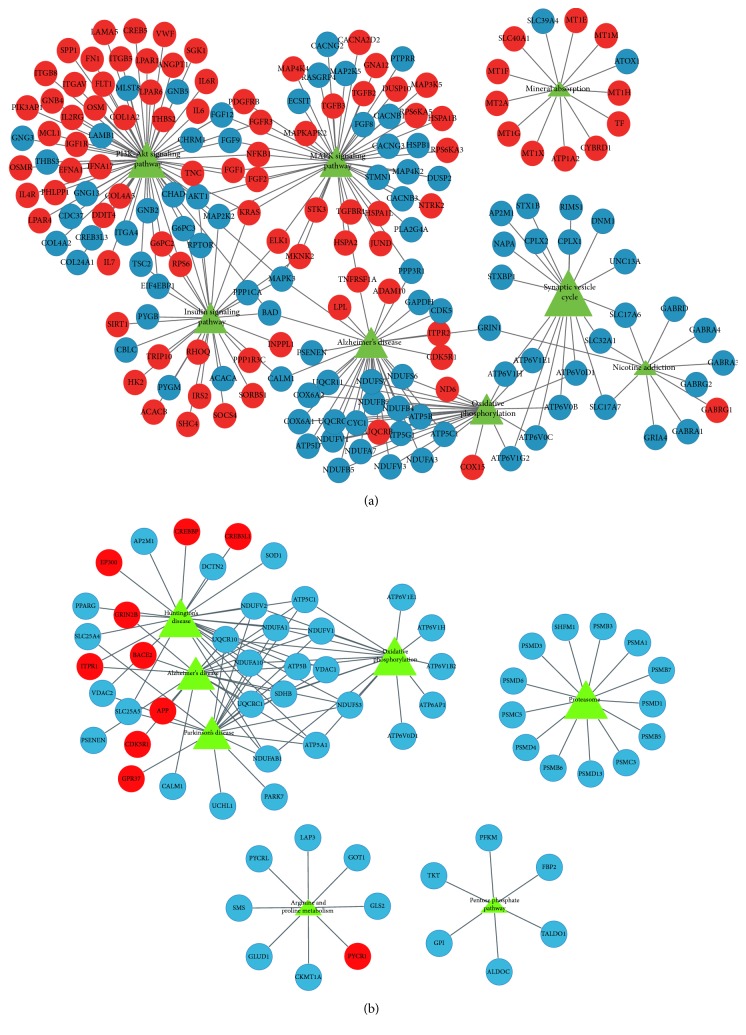
KEGG pathway enrichment of EC (a) and HIP (b) by DAVID. Red circle represents upregulated DEGs and blue circle represents downregulated DEGs.

**Figure 6:**
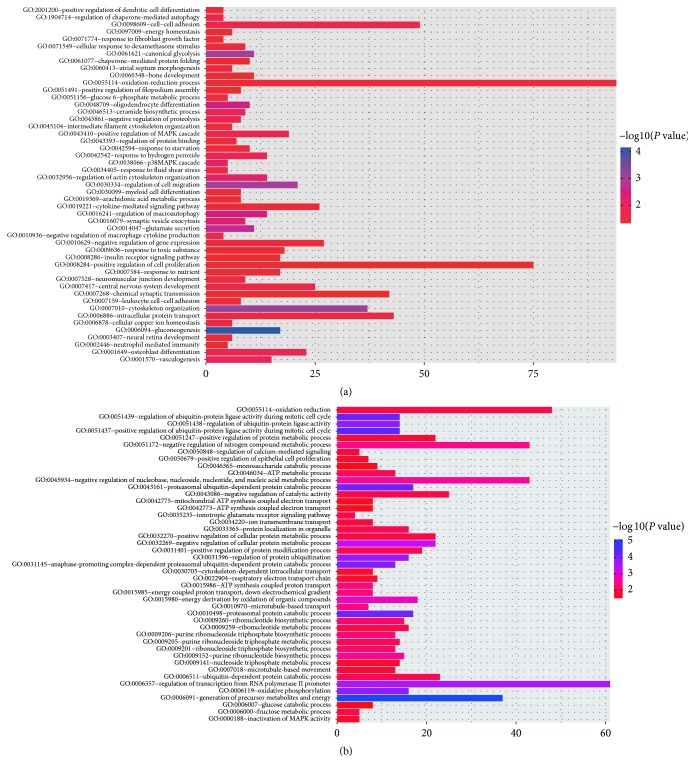
GO_BP enrichment of EC (a) and HIP (b) by DAVID. The *x*-axis represents the number of DEGs and the *y*-axis represents the GO_BP terms. The intensity of the color depends on the −log_10_ (*P* value).

To explore GO_BP and KEGG pathways in EC and HIP, we studied DEGs in these two regions using DAVID. A total of 97 GO_BP terms and 7 KEGG pathways dysregulated and 110 GO_BP terms and 7 KEGG pathways upregulated with *P* value < 0.05 were enriched in EC and HIP regions, respectively. The KEGG pathways and GO_BP terms were shown in Figures 5 and 6, respectively.

**Figure 7:**
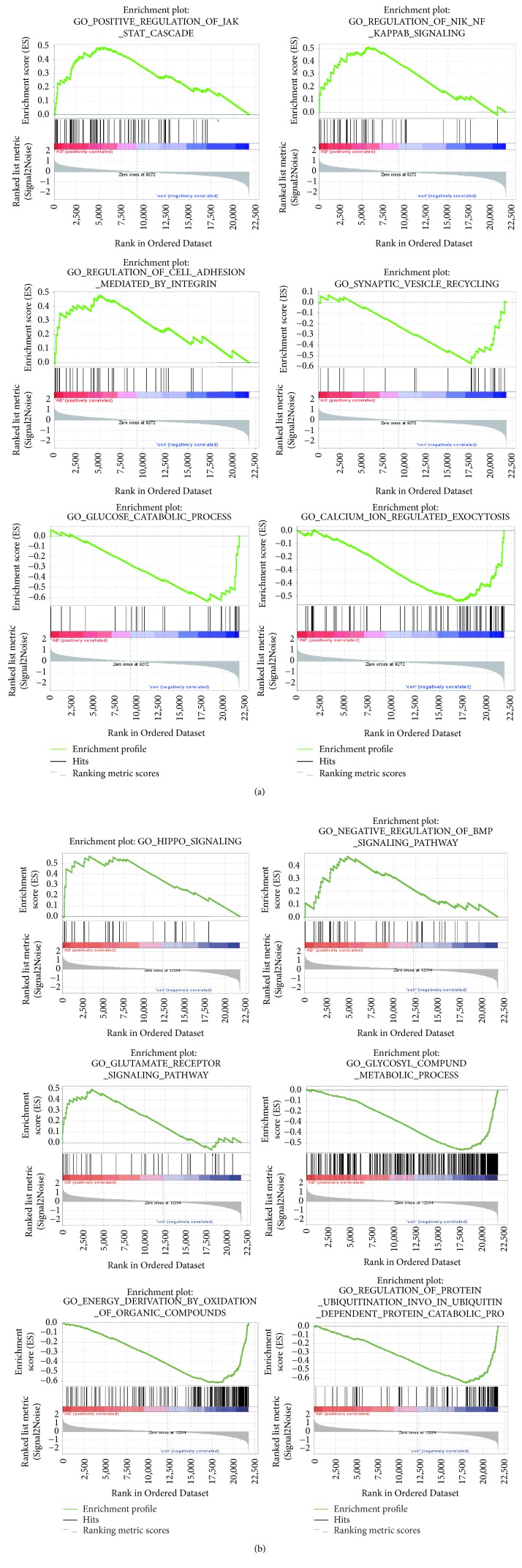
The top three representative enrichment plots of GO_BP of up- and downregulated pathways for EC (a) and HIP (b) were analyzed by GSEA.

DEGs in EC and HIP regions were also analyzed by the GSEA method which uses a database of several thousand predefined sets of genes. Genes in the same set share pathway or localization. GSEA is also able to detect small, but significant expression changes in these functionally connected genes that cannot be revealed by gene-by-gene comparisons. For EC, there were 10 gene sets with significant upregulation in AD compared to normal elderly control (*P* < 0.01) and 102 gene sets with significant downregulation (*P* < 0.01), respectively. For HIP, 6 gene sets showed significant upregulation in AD in comparison to normal elderly control (*P* < 0.01) while 341 gene sets were significantly downregulated (*P* < 0.01), respectively. The top three GO_BP terms of up- and downregulated pathways for EC and HIP were listed in Figure 7.

**Figure 8:**
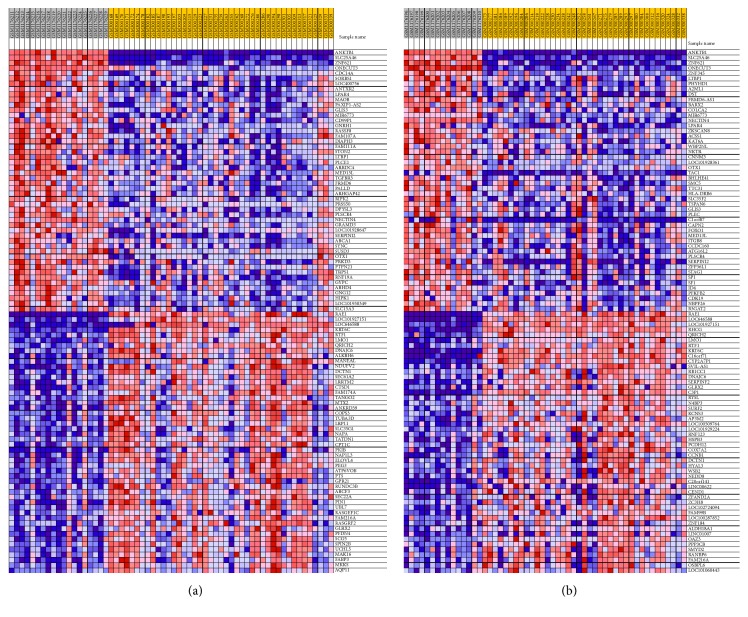
The heatmap of the top 50 features of EC (a) and HIP (b) for both AD and normal through analyzing the microarray profiles of GSE48350.

The microarray profiles of GSE48350 were also analyzed by GSEA. 1211 gene sets shown to be upregulated in AD EC region and 3 gene sets of them were significantly enriched at *P* < 0.01; 2327 gene sets were shown to be downregulated and 51 gene sets are significantly enriched at *P* < 0.01. There are 1494 gene sets upregulated in AD HIP region and 3 gene sets of them were significantly enriched at *P* < 0.01; 2044 gene sets are downregulated and 29 gene sets are significantly enriched at *P* < 0.01. The heat maps of the top 50 features in both AD and normal were shown in Figure 8.

### 3.4. Biomarkers in Blood

To find the potential biomarker in blood sample, firstly, we analyzed the DEGs of GSE4226 by Limma with the threshold for the *P* value < 0.05 and |log_2_⁡FC| > 1. Totally, 77 DEGs including 47 upregulated and 30 downregulated DEGs were identified. Some of them had the same variation trend as the DEGs in EC and HIP. For example,* CTSD* (Cathepsin-D) was downregulated, and* VCAM1* was upregulated significantly in blood, EC, and HIP. These DEGs in blood were enriched for GO biological pathway and KEGG pathway enrichment by DAVID. The dysregulated KEGG pathway included ribosome, cell adhesion molecules (CAMs), and tuberculosis. The GO_BP terms referred to SRP-dependent cotranslational protein targeting to membrane, rRNA processing, mitotic cell cycle checkpoint, antigen processing and presentation of exogenous peptide antigen via MHC class II, neural retina development, and regulation of transforming growth factor beta receptor signaling pathway.

**Figure 9:**
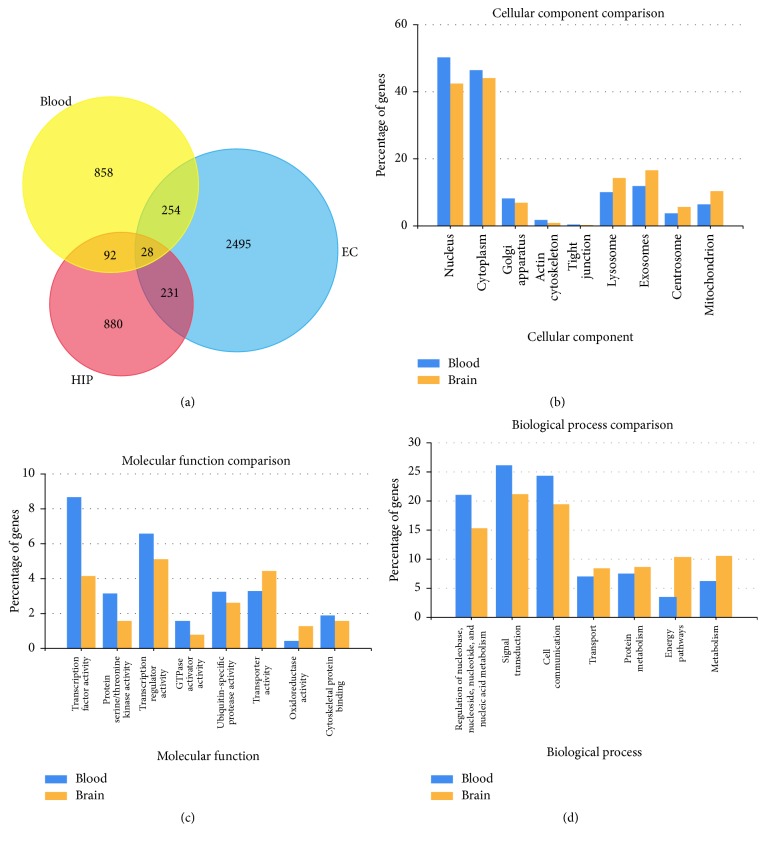
The predicted target genes of differently expressed microRNAs in blood compared with DEGs in EC or HIP by GO_terms using FunRich 2.1.2. (a) The Venn chart for overlapping DEGs of EC, HIP, and blood. (b) Comparison of cellular components. (c) Comparison of molecular functions. (d) Comparison of biological processes.

The significantly different miRNAs were extracted by Limma for GSE90828 microarray dataset and edgeR for GSE46579 RNA-seq dataset. The overlapping DEGs between these two datasets included has-let-7d, has-miR-144, has-miR-374a, and has-miR-106b. The target genes of the four miRNAs were predicted by targetScan and miRDB, and the intersection of both datasets for each miRNA was used for further study. KEGG pathway and GO_terms enrichment for the overlapping genes between target genes of the four miRNAs (has-let-7d, has-miR-144, has-miR-374a, and has-miR-106b) and DEGs in EC and HIP were shown in Table 1 and Figure 9.

### 3.5. Small Molecules Involved in AD

By WGCNA algorithm, we obtained the key nodes of coexpression network and the hub genes were divided into two groups of upregulated and downregulated genes. Using the two gene groups as input for the CMAP database, the six small molecules reversed with the dysregulation of AD in both EC and HIP were determined with *P* value < 0.05. Fisetin had a high score of −0.99, but the *P* value was not calculated for lack of enough assays (Table 2).

## 4. Discussion

Gene microarray technology has been applied to a variety of biological fields, such as molecular diagnosis, drug discovery, and pathogenic mechanism research because it was able to generate a lot of biological information. GEO database is an open access database with a great deal of gene expression data which is mainly derived from gene expression profiling studies [12]. Bioinformatics analytical software in combination with microarray technology provides a powerful approach for gaining insight into the hub genes, targets, and functional pathways related to AD.

### 4.1. Identification of Hub Genes from Gene Coexpression Network

Through coexpression network construction and analysis of node degree, we found some functional and high connected hubs varied in EC and HIP region, such as* CHRM1*,* MAPK1, TGFBR1*,* LIFR*,* ERBB4*,* ERBIN*,* ATP5C1*,* IGF1R*,* GJA1*,* TJP1*,* AP2M1*,* CDK5*,* FGF2*,* TUBB*,* SLC22A3*,* DBT*,* CDK13, NLN*, and* MIF*. Most of them have high enrichment score in GSE48350 analyzed by GSEA.

ErbB2 (Erb-B2 receptor tyrosine kinase 2) and ErbB4 (Erb-B2 receptor tyrosine kinase 4) are the important tyrosine kinases of ErbB system [29]. We found* ERBIN *and* ERBB4*, encoding ErbB2 and ErbB4, were significantly upregulated in EC region in GSE5281 and GSE48350 whereas the expression of neuregulin-1 is downregulated. Neuregulin-1 regulates developmental neuronal survival and synaptogenesis, astrocytic differentiation, and microglial activation [30]. Neuregulin-1 binds to ErbB4 and leads to a conformational change in ErbB4, which then dimerizes preferentially with ErbB2 [29, 30]. The formation of dimers leads to tyrosine phosphorylation and activates the corresponding downstream Akt and ERK signaling pathways, which regulate a variety of cell-specific functions, including neurogenesis, myelination, neuroinflammation, and neurotransmission [31]. Considering the dysregulation of PI3K-Akt pathway and MAPK pathway, ErbB2 and ErbB4 may be the potential targets for AD.

MIF (macrophage migration inhibitory factor), a proinflammatory cytokine, was identified as a binding partner of A*β* in vitro and observed in association with A*β* plaques within the human brain [32]. The expression of* GPI* (glycosylphosphatidylinositol) was significantly downregulated in both EC and HIP of AD. Previous studies have shown that* GPI* knockdown or mutation resulted in *α*-syn accumulation, neurotoxicity, neuroinflammatory signal, and induced neurodegeneration in Parkinson's models [33]. In addition, GPI-anchored proteins play important roles in protection against A*β*. Considering the significant change of* GPI* expression and the roles of GPI-anchored protein, GPI may be considered as therapeutic target in Alzheimer's disease [33].

In this study, we found* CDK13* (cyclin-dependent kinase 13) dysregulated remarkably in HIP region. CDK13 plays an important role in axonal elongation and regulation of CDK5 (cyclin-dependent kinase 5) expression [34]. Downregulated expression of CDK13 shortens the averaged axonal length and lower CDK5 expression. SLC22A3 (organic cation transporter 3, OCT3) is a low-affinity, high-capacity transporter widely expressed in the central nervous system (CNS) and other major organs in both humans and rodents. It is postulated that OCT3 has a role in the overall regulation of neurotransmission and maintenance of homeostasis within the CNS [35].

### 4.2. Dysregulated DEGs and Biological Pathway in EC and HIP

Some well-known AD-related DEGs were dysregulated in our study, such as* APP*,* CDK5R1*,* BACE2*,* PSENEN*,* GRIN2B*,* ADAM10*, and* TNFRSF1A*. According to GO_BP and KEGG pathway enrichment by DAVID, we also found DEGs referring to several pathways significantly dysregulated in EC and HIP, such as PI3K-Akt signaling pathway, MAPK signaling pathway, oxidative phosphorylation, synaptic vesicle cycle, cell-cell adhesion, cytokine-mediated signaling pathway, proteasome, arginine, and proline metabolism, and pentose phosphate pathway. The GSEA results were similar, including calcium ion regulated exocytosis, hippo signaling, BMP signaling, and glutamate receptor signaling pathway, in addition to the above-mentioned pathways.

There were some common DEGs varied in EC and HIP among AD, Huntington's disease, Parkinson's disease, and diabetes. An important property of the neurodegenerative diseases is the downregulated oxidative phosphorylation because of the dysfunctional brain mitochondria and the varied genes including* UQCRC1*,* UQCR10*,* SDHB*,* ATP5B*,* ATP5C1*,* NFUFA1*, and* VDAC1*. AD is a multifactor disease that has been reported to have a close association with type 2 diabetes (T2D) [36]. AKT1, significantly downregulated in EC, plays an important role in the relationship of AD and diabetes. Inflammation is a key pathological component in AD that has been proposed as a major mechanism in both the initiation and progression of the disease [37]. Plenty of inflammatory DEGs in EC were upregulated, such as* NFKB1*,* IL2RG*,* IL6*,* IL6R*,* IL4R*,* IL7*,* JUND*,* TGFBR1*,* TGFB2*, and* TGFB3*.

Neurotransmitter release is mediated by exocytosis of synaptic vesicles (SV) which undergoes a trafficking cycle [38]. In our study, the results showed that a series of genes associated with SV were downregulated, which would affect the amount of neurotransmitter available for release and contribute to synaptic degeneration. These genes included* STXBP1*,* NAPA*,* AP2M1*,* CPLX2*,* CPLX1*, and* STX1B*, which participate in exocytosis of vesicles loaded with a neurotransmitter, coordinated recovery of SVs by endocytosis, refilling of vesicles, and subsequent release of the refilled vesicles from the presynaptic bouton. These findings indicated potential roles of these key genes in synaptic communication in AD.

### 4.3. Biomarkers in Blood

Research has focused on identifying biomarkers for AD so that treatment can be carried out as soon as possible in order to restrict or prevent intellectual impairments, memory loss, and other cognitive abnormalities that are associated with the disease. Blood and blood components are primary sources for biomarkers, and miRNAs are known to be stable in the blood and blood components, serum and plasma [10]. Cathepsin-D (CTSD) and vascular cell adhesion molecule-1 (VCAM-1) were significantly dysregulated in blood, EC, and HIP. The miRNAs (has-let-7d, has-miR-144, has-miR-374a, and has-miR-106b) were significantly dysregulated in blood. Mutations in the* CTSD* are classically associated with severe congenital disease with microcephaly, cerebral and cerebellar atrophy, seizures, spasticity, central apnea, and death occurring in the first days of life [39, 40]. It was recently reported that* CTSD* is an excellent functional candidate AD gene that encodes a lysosomal aspartyl protease which degrades both amyloid ß and tau in vitro and a CTSD polymorphism may be associated with A*β* deposition in AD brain and to an increased risk of AD [41]. Here, we firstly found the expression of* CTSD* was downregulated in the blood of AD patients and CTSD was a potential blood biomarker for AD. VCAM-1 is a member of the immunoglobulin superfamily and is expressed by endothelial cells and elevated VCAM-1 levels might reflect defects of the vascular system [42]. However, a significant association between age and VCAM-1 independent of the cardiovascular risk was shown, and others also found VCAM-1 elevated in plasma of AD cases [43], which is consistent with our findings.

### 4.4. Crosstalk between Blood and Brain

To seek for the mechanism behind a crosstalk between peripheral blood and central nervous system, we compared the target genes of the four miRNAs (has-let-7d, has-miR-144, has-miR-374a, and has-miR-106b) with DEGs in EC and HIP regions. There were 28 genes in the intersection part of blood, EC, and HIP (Figure 9(a)). These genes includes* AKAP13, AMER2, BBX, CEP97, CYP46A1, DPP3, DUSP2, GLIS3, GNB2, KLHL31, KMT2E, L1CAM, MINK1, NLN, PCSK5, PFKFB3, PHF6, PRPF38B, RB1, RGS7BP, RSF1, SEMA4C, SLC35F3, SLITRK2, SNX17, TNKS1BP1, TNPO1*, and* ZBTB41*. Most of these genes had a potential relationship with central nervous system. For example,* NLN *(Neprilysin) is a high connected hub gene varied in EC and HIP region, which has a close relationship with the clearance of A*β* [44]. Like VCAM-1 mentioned above, L1CAM (L1 cell adhesion molecule) is another cell adhesion molecule with an important role in the development of the nervous system [45]. Through analyzing the target genes and DEGs in the union set of EC and HIP, we found that these target genes had a similar GO_terms distribution to EC and HIP (Figures 9(b), 9(c), and 9(d)). Cellular component described that these genes were prevailingly located in nucleus, cytoplasma, exosome, and lysosome. Several studies have suggested the multifaceted roles of exosomes in AD [46]. Exosomes derived from the central nervous system can be found in peripheral fluids, which suggested the existence of a crosstalk between blood and brain. The enrichment of molecular function was consistent with that of cellular component, mainly referring to transcript factor activity, ubiquitin-specific protease activity, and transport activities. Based on GO_BP and KEGG pathway enrichment analysis (Table 2), we found genes related to MAPK signaling pathway, insulin signaling pathway, axon guidance, focal adhesion, and energy pathways dysregulated in blood and brain, such as* AKT1*,* TGFBR1*,* IGF1R*,* EPHA5*,* VCAM1*, and* L1CAM*. Therefore, we speculated that the four miRNAs might build a bridge between peripheral blood and central nervous system and were the potential biomarker for AD diagnosis.

### 4.5. Predicted Small Molecules for AD Treatment

Six small molecules, which were most relevant to the degenerative AD, were screened out based on the downregulated and upregulated genes. Fisetin (3,3′,4′,7-tetrahydroxyflavone) had the highest enrichment score, but the *P* value could not be calculated without enough samples. Fisetin has a strong anti-inflammatory activity in brain microglia and has been found to be neuroprotective, induce neuronal differentiation, enhance memory, and inhibit the aggregation of A*β* that may cause the progressive neuronal loss in AD [47]. The GSK3*β* (glycogen synthase kinase 3 beta) inhibitor, alsterpaullone, was found to suppress toxicity of tau in a concentration-dependent manner [48]. Telenzepine, an M1-selective muscarinic receptor antagonist, can prevent the induction of a long-lasting excitatory postsynaptic potential in autonomic ganglia [49]. Bezafibrate (BEZ), the pan-PPAR (peroxisome proliferator-activated receptor) activator, is commonly used to treat dyslipidemia [50]. Recent research finds that PPARs can significantly reduce tau protein level and microglia activation, promote mitochondrial biogenesis, and improve behavioral activities in P301S mice [51]. In COX10 knockout mice, bezafibrate increases mitochondrial ATP synthesis and decreases astrocyte proliferation and inflammatory factors,suggesting that bezafibrate has a potential significance in the treatment of neurodegenerative diseases [52]. Scoulerine which is isolated from* Corydalis cava* (Fumariaceae), used in folk medicine in the treatment of memory dysfunctions, was found to be active as BACE1 (beta-secretase 1) inhibitor [53]. Melatonin (*N*-acetyl-5-methoxytryptamine) is an endogenous neurohormone whose level decreases during aging, especially in AD patients. It had been reported to possess strong antioxidant property and is able to directly scavenge a variety of reactive oxygen species [54]. Moreover, it has been demonstrated to be a moderate inhibitor of A*β* aggregation.

In conclusion, we found some functional hub genes, whose encoding protein has a close relationship to AD treatment, such as* ErbB2*,* ErbB4*,* OCT3*,* MIF*,* CDK13*, and* GPI*. Several pathways were significantly dysregulated in EC and HIP, such as PI3K-Akt signaling pathway, MAPK signaling pathway, insulin signaling pathway, oxidative phosphorylation, synaptic vesicle cycle, cell-cell adhesion, proteasome, arginine, and proline metabolism, pentose phosphate pathway, calcium ion regulated exocytosis, and glutamate receptor signaling pathway.* CTSD* and* VCAM1* were dysregulated significantly in blood, EC, and HIP, which were potential biomarkers for AD. From the target genes of the four miRNAs and DEGs in AD brain, we found inflammation, defective insulin signaling, and energy metabolism linked the pathogenesis of peripheral and central nervous system. Last, based on the hub genes in EC and HIP, six small molecules were screened out and they were fisetin, alsterpaullone, telenzepine, bezafibrate, scoulerine, and melatonin, which had direct or indirect relationships with the treatment of memory dysfunction. These biological pathways and DEGs or hub genes will help to elucidate AD pathogenesis and identify novel biomarkers or drug targets for developing improved diagnostics and therapeutics against AD.

## Figures and Tables

**Table 1:** KEGG pathway enrichment for the overlapping genes between target genes of the four microRNAs (has-let-7d, has-miR-144, has-miR-374a, and has-miR-106b) and the DEGs in EC and HIP regions.

Term	Count	*P* value	Genes
MAPK signaling pathway	13	0.0062	RPS6KA5, AKT1, MAP4K4, MAP3K5, PLA2G4A, DUSP2, FGF9, TGFBR1, MAP2K4, MKNK2, PPP3R1, FGF12, DUSP6
Endocytosis	10	0.010	IGF1R, ADRB3, FLT1, TGFBR1, PSD3, VPS4B, DNAJC6, PSD2, ARAP2, LDLRAP1
Axon guidance	7	0.042	EPHA5, SEMA6A, PLXNA1, PPP3R1, SEMA4C, L1CAM, SRGAP1
Insulin signaling pathway	6	0.0076	AKT1, L1CAM, IGF1R, IRS2, SOCS7, RHOQ
Focal adhesion	4	0.021	VCAM1, L1CAM, ITGA4, CERCAM

**Table 2:** Small molecules which might reverse the dysregulation of AD in EC and HIP regions.

Cmap name	Score	*P*
Alsterpaullone	−0.93	0.00052
Telenzepine	−0.82	0.0030
Bezafibrate	−0.81	0.0043
Scoulerine	−0.78	0.0067
Melatonin	−0.78	0.027
Fisetin	−0.99	—
